# GM trees with increased resistance to herbivores: trait efficiency and their potential to promote tree growth

**DOI:** 10.3389/fpls.2015.00279

**Published:** 2015-05-01

**Authors:** Joakim Hjältén, E. Petter Axelsson

**Affiliations:** ^1^Department of Wildlife, Fish, and Environmental Studies, Swedish University of Agricultural SciencesUmeå, Sweden; ^2^Department of Forest Ecology and Management, Swedish University of Agricultural SciencesUmeå, Sweden

**Keywords:** GM trees, herbivore resistance, traits efficiency, tree growth, leaf damage

## Abstract

Climate change, as well as a more intensive forestry, is expected to increase the risk of damage by pests and pathogens on trees, which can already be a severe problem in tree plantations. Recent development of biotechnology theoretically allows for resistance enhancement that could help reduce these risks but we still lack a comprehensive understanding of benefits and tradeoffs with pest resistant GM (genetically modified) trees. We synthesized the current knowledge on the effectiveness of GM forest trees with increased resistance to herbivores. There is ample evidence that induction of exogenous *Bacillus thuringiensis* genes reduce performance of target pests whereas upregulation of endogenous resistance traits e.g., phenolics, generates variable results. Our review identified very few studies estimating the realized benefits in tree growth of GM trees in the field. This is concerning as the realized benefit with insect resistant GM plants seems to be context-dependent and likely manifested only if herbivore pressure is sufficiently high. Future studies of secondary pest species and resistance evolution in pest to GM trees should be prioritized. But most importantly we need more long-term field tests to evaluate the benefits and risks with pest resistant GM trees.

## Introduction

**Figure 1:**
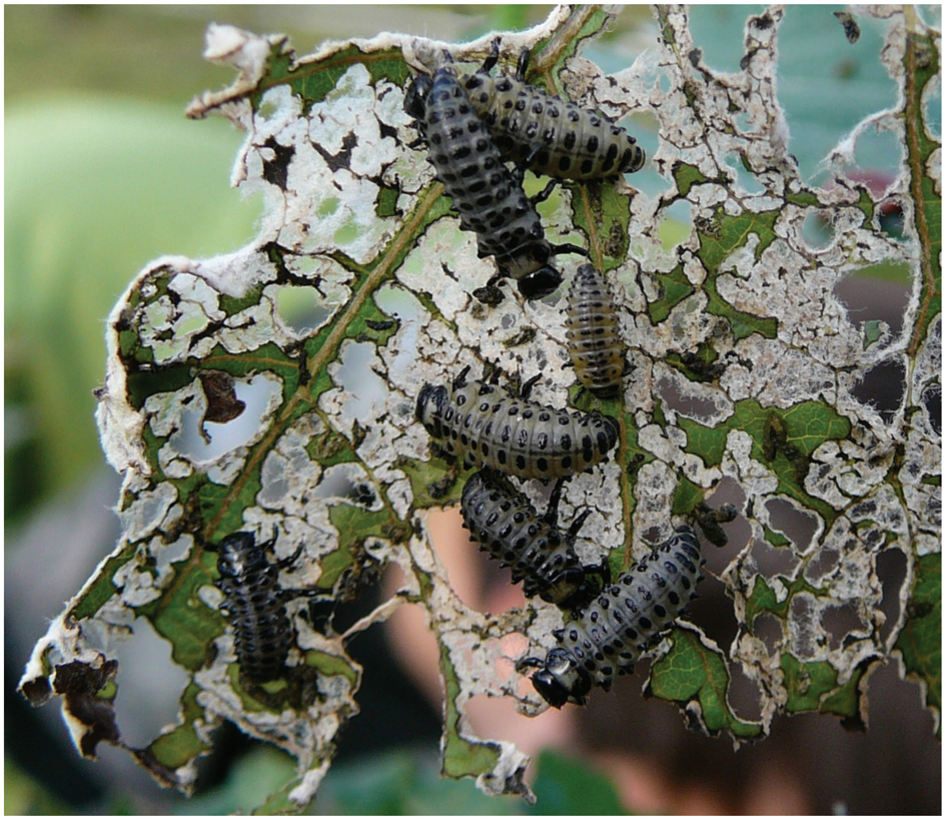
**Damage by leaf beetle larvae in an aspen plantation**.

There is an urgent need to find alternatives to fossil fuels to reduce our input of CO^2^ into the atmosphere and thus mitigate climate change. It has been estimated that trees can become a major source of bioenergy and help mitigate the anticipated rise in CO^2^ over the next 50 years (Smeets and Faaij, 2007). This may require more intense management practices, such as the use of trees as short rotation energy crops. However, intensified forestry is expected to increase the risk of damage by pests and pathogens on trees (Klapwijk et al., 2013). Pests can already be a severe problem in tree plantations (Gruppe et al., 1999) and pest problems are expected to increase due to ongoing climate change (Klapwijk et al., 2013) (Figure 1). Tree pests can severely effect growth and survival of forest trees and thus inflict large economic losses (Ayres and Lombardero, 2000).

In general only a few herbivores on a particular tree species develop into pests. For example, only a small number of Populus-feeding insects are regarded as serious pests in North America. But these insects can severely hamper establishment, reduce growth, and increase mortality in managed stands (Dickmann and Stuart, 1983; Coyle et al., 2002; Nordman et al., 2005). Important pests on conifers in North America include both defoliating insects (e.g., pine sawflies) and cambium consumers (bark beetles) (Dukes et al., 2009). In China, damage to hybrid Populus plantations by the poplar lopper (*Apochemia cinerarua*) and the gypsy moth (*Lymantria dispar*) can inflict substantial (up to 40%) stand losses (Hu et al., 2001). Similarly, in Europe Gruppe et al. (1999) reported up to 50% leaf damage on some aspen clones and that a large majority of damage was inflicted by a few but abundant pest species, such as the leaf beetles *Chrysomela tremulae* and *Phratora vitellinae*, and the leaf roller *Byctiscus populi* (Curculionidae: Coleoptera). Coyle et al. (2002) found that leaf beetles damage reduced above ground biomass of aspens by 50–73%. Bark beetles, pine weevils, and pine moths are important pests on conifers in Europe and the impact of pests on forests are expected to increase in a warming climate (Leather et al., 1999; Jacquet et al., 2012; Marini et al., 2012).

The expected increasing impact of pests could uncontrolled cause severe problems in the form of reduced production, lower economic yield, and profoundly reduce our ability to replace fossil fuels with bioenergy. It could also result in increased use of pesticides which could have negative effects on the environment. It is therefore of outmost importance that we find other means to control pests and pathogens on trees. This likely calls for developments in a range of fields including changes to management practices and technology but also improvements in tree characteristics through genetic enhancements (Smeets and Faaij, 2007; Fenning et al., 2008).

With the recent developments in biotechnology, genetic enhancement of trees theoretically allows modification of most individual traits in selected genotypes. As a result, GM (gene modification) technology is much more specific and is not hampered by the constraint of traditional breeding, e.g., late flowering, slow maturation, long reproductive cycles, and complex mating systems (including self-incompatibility and a high degree of heterozygosity) in trees. Difficulties in identifying the best parents (and controlling their mating), maintaining genetic gain with high heterozygosity (Cheliak and Rogers, 1990), and understanding the complex genomes of many tree species causes problems for tree breeders. Consequently, tree breeding is a slow process that generally, in a short time span, only allows for minor improvements of tree resistance (Ye et al., 2011). By contrast, through genetic engineering, it is possible to introduce novel traits as well as regulate native traits and thus change plant expression of various biochemicals, which allow new strategies for breeding (FAO, 2004).

Two principally different strategies are used to modify plants for enhanced resistance. The first is to up-regulate innate resistance traits (endogenous traits) such as phenolics and the second is to introduce new traits (exogenous traits) such as the production of *Bacillus thuringiensis* (hereafter Bt) toxins. The principal difference between these two approaches is that endogenous traits are something that herbivores have evolved mechanisms to deal with, e.g., some specialists use plant derived chemicals for their own defense (Pasteels et al., 1983). Typically these defenses work as a deterrent or by reducing growth rate of herbivores. Exogenous traits on the other hand are completely novel to the herbivores and their inherent ability for resistance are expected to be restricted (but see for example Genissel et al., 2003) and exogenous traits often cause high mortality in targeted pests. Thus, these two GM approaches can be expected to have different effects on herbivore guilds.

However, even if GM trees with increased resistance to herbivores have been available for more than 25 years, we still lack knowledge on the efficiency of different modifications for resistance in reducing damage by forest pests and thereby enhancing tree growth. Lab studies evaluating resistance are relatively common but field studies are rare (Ye et al., 2011). Still, before a commercial release of GM trees, it is important that benefits and risk are balanced and for that we need a proper understanding of the benefits as well as the risks of GM trees.

## Aim

The aim of this study was to synthesize the current knowledge on the effectiveness of GM forest trees (fruit trees are not included in our study) with increased resistance to herbivores. More specifically we ask how effective different types of enhanced resistance are at reducing damage by pests and how well this translates into increased growth and production in GM trees. In addition, based on the above synthesis we highlight areas of uncertainty and suggest future directions for resistance enhancement in GM trees.

To collect the necessary literature we used the following search string in Web of Science: Tree and [(Transgenic, GMO, GE, or GM) and (resistance) and (herbivores)]. Selected articles included studies on preference and performance of herbivores, and damage on plants and effects on plant performance in terms of growth and biomass production.

## Current knowledge

Populus became the first genera to be genetically transformed and regenerated (Fillatti et al., 1987) and it now serves as the leading model system in forest biotechnology (FAO, 2010; Ye et al., 2011). Ye et al. (2011) reviewed different applications of genetic modifications in Populus and showed that in this single tree taxon, modified traits include; insect- herbicide- and disease-resistance, tolerance toward different abiotic stresses, wood properties, and growth (i.e., growth-rate, rooting, and flowering). In general, field studies with GM forest trees are restricted to a few species, with Populus species clearly dominating (Häggman et al., 2013). Other genera studied in field experiments include Pinus, Liquidambar, and Eucalyptus, and, more recently, Picea, and Betula (Häggman et al., 2013). The majority of field studies address questions related to methods (e.g., gene stability, gene expression) or traits related to herbicide resistance, wood quality, or wood chemistry and very few deal with pest resistance (FAO, 2010; Häggman et al., 2013). Our review on resistance in GM trees revealed only three “true” field studies and one study conducted with potted plants in “semi-natural” environments and all of these addressed pest resistance in *Populus* sp.

## Effects in target and non-target pests

The singly most common transformation for pest resistance involves the introduction of exogenous Bt genes, enabling the plant to produce Cry toxins lethal to certain targeted insect pests. More than 150 different Cry proteins have been identified (Schnepf et al., 1998), with examples including Cry3Aa proteins targeting coleopteran insects and the Cry1 and Cry2 families effective against lepidopteran species (Hu et al., 2001; Hussein et al., 2005). Other exogenous defenses include scorpion neurotoxin (Ye et al., 2011) and tobacco anionic peroxidase (Dowd et al., 1998).

The effectiveness of Bt toxins against specific pest species on trees has been tested and verified both in laboratory (Genissel et al., 2003; Kleiner et al., 2003) and field studies (Hu et al., 2001; Ye et al., 2011). Most studies report significant reduction of consumption and performance of target insect pests on Bt trees. Genissel et al. (2003) found that ingestion of leaves from Bt aspens induced 100% mortality in *Chrysomela tremulae* within 2–13 days depending on instar, with older instars and adult beetles living the longest. Bt induction in *Pinus radiata* induced up to 80% mortality in larvae of the painted apple moth, *Teia anartoides* (Grace et al., 2005). Similar results have been found in other studies of both conifers and deciduous trees (Shin et al., 1994; Kleiner et al., 1995; Harcourt et al., 2000; Tang and Tian, 2003; Lachance et al., 2007; Zhang et al., 2011) (Table 1). Thus, there is ample evidence that Bt have a strong impact on survival and performance of target pests and that even short time Bt exposure can cause long lasting effects (Hjälten et al., 2013). However, Axelsson et al. (2012) found that the density of one species from the targeted order, the leaf rolling beetle *Byctiscus populi* (Coleoptera), was unaffected on Bt aspens. Furthermore, non-target herbivores belonging to different genera than the target pests seem unaffected by Bt plants (Ye et al., 2011; Zhang et al., 2011) or even prefer them (Axelsson et al., 2011). Induction of exogenous tobacco anionic peroxidase in GM *Liquidambar styraciflua* had variable effects on the herbivore guild. It resulted in negative effects on the preference/performance of some herbivores but others preferred GM trees (Dowd et al., 1998).

**Table 1:** **Summary of studies addressing herbivore preference/performance on GM trees and/or GM plant performance in the presence of herbivores**.

**Focal tree**	**Modification aimed at**	**Gene construct**	**Genome**	**Environmental setting and pest species studied**	**Response variable (s)**	**Herbivore response to GM trees compared to wild-type control trees**	**GM plant response compared to wild-type control trees**	**References**
*P. tremula x P. tremuloides*	Insect resistance	Overexpression of Bt	Exogenous	Field semi-natural/damage by naturally occurring insect herbivores	Herbivore leaf damage and tree biomass production	Significant decrease in leaf damage	Ns	Axelsson et al., 2012
*P. tremula x P. tremuloides*	Insect resistance	Overexpression of Bt	Exogenous	Greenhouse/slugs	Leaf damage	Significant increase in leaf damage		Axelsson et al., 2011
*Liquidambar styraciflua*	Insect resistance	Overexpression of tobacco anionic peroxidase	Exogenous	Lab/*Malacosoma disstria, Lymantria dispar, Spodoptera frugiperda Helicoerpa zea and Ostrinia nubilalis*	Preference/performance	Significant increase or decrease (depending on herbivore species) in feeding rate and survival		Dowd et al., 1998
*Pinus radiata*	Insect resistance	Overexpression of Bt	Exogenous	Lab/*Teia anartoides*	Larval mortality	Significantly increased mortality		Grace et al., 2005
*Eucalyptus camaldulensis*	Insect resistance	Overexpression of Bt	Exogenous	Lab/chrysomelid beetles	Leaf feeding and leaf beetle survival	Significant decrease in both variables		Harcourt et al., 2000
*P. tremula x P. tremuloides*	Insect resistance	Overexpression of Bt	Exogenous	Lab/chrysomelid beetles	Leaf feeding and plant growth	Significant decrease in leaf feeding	Significant increase in plant height	Hjältén et al., 2012
*Populus nigra*	Insect resistance	Overexpression of Bt	Exogenous	Field/naturally occurring herbivores	Leaf damage	Decrease in leaf damage		Hu et al., 2007
*Populus nigra*	Insect resistance	Overexpression of Bt	Exogenous	Field/naturally occurring herbivores	Leaf damage and abundance of insect larvae	Decrease in larval density		Hu et al., 2001
*P. alba L. x P. grandidentata*	Insect resistance	Overexpression of Bt	Exogenous	Lab/*Malacosoma disstria and Lymantria*	Consumption and performance	Decrease in herbivore both variables		Kleiner et al., 1995
*P. alba L. x P. grandidentata*	Insect resistance	Overexpression of Bt	Exogenous	Lab/*Lymantria dispar*	Consumption and performance	Decrease in both variables		Kleiner et al., 2003
*P. deltoides x P. nigra*	Insect resistance	Overexpression of Bt	Exogenous	Field/Naturally occurring herbivores	Insect damage and plant growth	Significant decrease in damage	Significant increase in plant growth	Klocko et al., 2014
*Picea glauca*	Insect resistance	Overexpression of Bt	Exogenous	Lab and field/*Choristoneura fumiferana*	Larval mortality	Significantly increased mortality		Lachance et al., 2007
*Larix decidua*	Insect resistance	Overexpression of Bt	Exogenous	Lab/*Lymantria dispar*	Insect growth and needle damage	Significant decrease in growth and needle damage		Shin et al., 1994
*Pinus radiata*	Insect resistance	Overexpression of Bt	Exogenous	Lab/*Dendrolimus punctatus* and *Crypyothelea formosicola*	Survival and consumption	Decreased survival and consumption		Tang and Tian, 2003
*Populus alba x P. glandulosa*	Insect resistance	Overexpression of Bt	Exogenous	Lab/*Plagiodera versicolora* (target) and *Clostera anachoreta* (non-target)	Insect mortality and pupation rate	Significantly increased mortality of *Plagiodera versicolora*		Zhang et al., 2011
*Populus tremula x P. alba*	Insect resistance	Overexpression of Ascorbate oxidase	Endogenus	Greenhouse/*Lymantria dispar and Melanoplus sanguinipes*	Leaf consumption and insect growth rate	Ns		Barbehenn et al., 2008
*P. tremula x P. tremuloides*	Insect resistance	Overexpression of the MYB134 tannin regulatory gene	Endogenus	Lab/*Malacosoma disstria* and *Lymantria dispar*	Preference/performance	Significant increase in both variables		Boeckler et al., 2014
*Populus tremuloides*	Improved pulping performance	4-Coumarate: coenzyme A ligase (4CL)	Endogenus	Greenhouse/*Lymantria dispar* and *Malacosoma disstria*	Preference/performance	Significant decrease in one of four lines		Brodeur-Campbell et al., 2006
*P. tremula x P. alba*	Insect resistance	Overexpression of *Camptotheca acuminata* tryptophan decarboxylas	Endogenous	Lab/*Malacosoma disstria*	Preference/performance	Significant decrease in growth rate		Gill and Ellis, 2006
*P. tremula x P. tremuloides*	Plant growth	Overexpression of sucrose phosphate synthase	Endogenous	Lab/chrysomelid beetles	Leaf chemistry and leaf feeding	Significant decrease in one of two lines coupled to an increase in phenolic concentration		Hjältén et al., 2007a
*P. tremula x P. tremuloides*	Plant growth	Overexpression of sucrose phosphate synthase	Endogenous	Lab/voles	Shoot feeding	Ns		Hjältén et al., 2008
*Populus tremula x P. alba*	Insect resistance	Overexpression of Leaf polyphenol oxidase	Endogenous	Lab/*Malacosoma disstria*	Insect growth, mortality and preference	Significant decrease in growth rate and increased mortality but no effect on feeding preference		Wang and Constabel, 2004
*(P. tometosa x P. bolleana) x P. tomentosa*	Insect resistance	Overexpression of cowpea trypsin inhibitor	Endogenous	Lab/*Malacosoma disstria, Lymantria dispar* and *Stilpnotia candida*	Insect growth, mortality and leaf consumption	Generally increased mortality and decreased growth and consumption		Zhang et al., 2005

Up-regulation of endogenous defenses in GM trees include e.g., different types of plant phenolics, that are known to act as plant defenses against tree pests (Miranda et al., 2007; Barbehenn and Constabel, 2011). GM trees with overexpression of condensed tannins, proteinase inhibitor (Ye et al., 2011), leaf polyphenol oxidase (Wang and Constabel, 2004) and trypsin inhibitors (Zhang et al., 2005) has therefore been produced. Although up-regulation of endogenous defenses can be effective, e.g., expression of tryptophan decarboxylase gene in aspens reduced the growth rate of *Malacosoma disstria* (Gill and Ellis, 2006), the effects may not always be consistent. Boeckler et al. (2014) reported that up-regulation of condensed tannins synthesis increased performance and leaf consumption by *Malacosoma disstria* and *Lymantria dispar*. This could potentially be explained by reduced levels of phenolic glucosides in GM lines (a consequence of the up-regulation of condensed tannin synthesis) as phenolic glucosides can deter herbivores (Boeckler et al., 2014). Furthermore, increased synthesis of ascorbate oxidase in GM aspen had no significant effect on insect pests (Barbehenn et al., 2008).

Thus, the outcome of induction of exogenous such as Bt on pest resistance seem more consistence than induction of endogenous traits. However, one reason for this pattern could be that modifications of endogenous traits on pest resistance have been much less studied than the effects of e.g., Bt induction. Thus, more studies on enhancement of endogenous defense traits, including indirect defenses (e.g., traits attracting natural enemies to herbivores) are urgently needed. Still, potential drawback with the use of endogenous defenses is the intraspecific variation in insect adaptation to endogenous plant defenses (Pentzold et al., 2014). Also, coevolution between certain herbivores and their host may cause defensive substances previously working deterrent to the herbivore to act as feeding stimulants (Hjältén et al., 2007b). Consequently specialist and generalist herbivores may respond differently to changes in the expression of plant defenses (Hjältén et al., 2007b; Boeckler et al., 2011). Potential drawback with exogenous traits like Bt is evolution of Bt resistance in target pests (Tabashnik et al., 2013). In addition, due to the high specificity of Bt, outbreaks of secondary non-targets pest could become a severe problem (Dorhout and Rice, 2010). There are also indications that Bt and tannins interact antagonistically with respect to herbivore resistance, i.e., that tannins reduced the negative effect of Bt on the performance and survival of spruce budworm (Bauce et al., 2006). However, by contrast (Guan et al., 2009; Delvas et al., 2011) found synergistic effects of tannins and Bt on *Helicoverpa armigera*, an important pest on cotton. Thus, the combined effect of exogenous and endogenous defense traits is unclear.

Careful examination of indirect metabolic changes in transformed lines is also important for proper assessment of benefits and risks. Inconsistencies in how pests respond to genetic modifications may stem from unintended changes in other traits of importance for plant resistance. Events in the transformation process may cause variability in gene expression or gene silencing and have secondary, unintended effects on plant physiology and innate resistance traits. Transformations intended to increase growth or improve pulping performance can induce unintended effects on tree resistance to pests and pathogens (Brodeur-Campbell et al., 2006; Hjältén et al., 2007a). As unintended effects could have both positive and negative effects on plant resistance, they need to be considered in evaluation of GM trees (Brodeur-Campbell et al., 2006; Hjältén et al., 2007a; Axelsson et al., 2011).

## Realized benefits in tree growth

Although both field-and lab experiments suggest a high efficiency of GM resistance traits against target herbivores (Hu et al., 2001; Balestrazzi et al., 2006), our review identified very few studies estimating the realized benefits in tree growth (Table 1). Significant reduced leaf damage on Bt trees have been found in both lab and greenhouse studies (Harcourt et al., 2000; Hjältén et al., 2012) as well as in field studies (Hu et al., 2001; Axelsson et al., 2012). This sometimes translates to higher growth in Bt plants (Hjältén et al., 2012; Klocko et al., 2014) but this is not always the case (Axelsson et al., 2012). One likely explanation for the lack of growth response could be that the damage levels are too low to induce reduced growth in wildtype plants and under such circumstances Bt induction provides no growth benefits (Axelsson et al., 2012). Leaf damage levels by insects in aspen plantations range between 3.8% and ca 50% (Gruppe et al., 1999; Coyle et al., 2002; Tomescu and Nef, 2007). The damage level in Axelsson et al. (2012) was only ~3.5%, probably too low to have any significant impact on tree biomass growth. A plants ability to compensate for herbivory damage depends both on the type and timing of damage as well as the nutritional status of plants (Hjältén et al., 1993; Anttonen et al., 2002) and young trees can compensate for 25% leaf loss if well fertilized (Anttonen et al., 2002). Thus, the realized benefit with insect resistant GM plants is context-dependent and is likely to be manifested only if herbivore pressure is sufficiently high (Hjältén et al., 2012). Klocko et al. (2014) reported in excess of 25% leaf damage on wildtype aspen, and as a likely consequence of the high herbivore pressure, they found 14% higher biomass in Bt aspens compared to the wildtype under field conditions.

## Knowledge gaps

From this short review it is clear that we need more field studies and especially long-term studies of growth benefits of pest resistant GM trees. Often the effects in controlled environments such as the lab or greenhouse cannot be extrapolated to natural environments. Also, when time is scaled up, predictability is reduced (Raffa, 2001). Without knowledge of long-term benefits of GM trees under different growing conditions, it is impossible to conduct proper cost-benefit analyses. However, our ability to conduct long term field experiments are often limited by the strict regulation for field trials with GM trees (Häggman et al., 2013). The study by Klocko et al. (2014) is one of the few field studies of realized benefits spanning more than one field season. Given that trees are long lived organisms associated with a myriad of species, the circumstances faced by a tree throughout its growing cycle may reduce our ability to predict the realized effect in the field.

Secondary pest species could reduce the benefits of insect resistant GM trees. Raffa (1989) suggested that the effective prevention of dominant pest species should benefit less dominant natural enemies. Experiences from crop systems support this prediction, Dorhout and Rice (2010) demonstrated enhanced survival of, and a corresponding range shift in Western bean cutworm (*Striacosta albicosta*) foraging on Bt corn lacking the target pest species European corn borer (*Ostrinia nubilalis*) and corn earworm (*Helicoverpa zea*). Similarly, Lu et al. (2010) found progressive increased population sizes of Mirid bugs in cotton and other crops, associated with a regional increase in Bt cotton which was adopted to target cotton bollworm (*Helicoverpa armigera*). Thus, secondary pest species could develop into a major problem in GM tree systems and strategies to counteract such development should be developed.

Resistance evolution in pests is a topic of debate which may be of special concern when it comes to long lived crops such as trees. Resistance evolution in pests and weeds toward different pesticide treatments are not uncommon (Tong et al., 2013) and the number of examples of pests evolving resistance toward insect resistant GM plants expressing Bt toxins are also growing. Tabashnik et al. (2011) reported reduced efficiency of Bt crops caused by field-evolved resistance in 5 out of 13 examined pest species. For trees, the poplar leaf beetle *Chrysomela tremulae* rapidly evolved resistance to sprays of Bt toxins in the laboratory and in the field (Augustin et al., 2004), which underscores that resistance development remains a serious concern and stresses the need for resistance management of Bt plants.

Most studies of resistance in GM trees have been conducted on deciduous trees. However, many of the most commercially important forest trees are conifer. Furthermore, many of the pest species on conifers are not leaf-feeders but rather cambium feeders, e.g., bark beetles. It is unclear how they might respond to induction of resistance traits in growing tissue, such as leaves, because other defense mechanisms, such as resin flow, confer resistance against these types of herbivores (Boone et al., 2011). Thus, more focus should be on evaluating the benefits of different types of GM conifers with respect to pest resistance.

## Ways forward

The knowledge gaps identified in this review involves the efficiency of upregulated endogenous defenses on pest species. As some specialist pest species are adapted to deal with some types of plant defenses and even use them as defenses themselves, their response to resistance enhancements might not be the expected (Pentzold et al., 2014). This also has implications for traditional resistance breeding and explains why this method only allows for minor improvements of tree resistance. In that respect, exogenous resistance traits hold better promises of, at least initially, higher efficiency. However, the problem with, e.g., Bt is that it targets specific taxa, and this infers a risk of secondary pest outbreaks. Thus, a combination of induction of both exogenous and endogenous resistant traits in GM trees might be advantageous as it might reduce the risk for outbreaks of secondary pests, as can be inferred from studies in crop systems (Ma et al., 2014). The development of GM technology for increased pest resistance likely need to be tackled on a broad front that includes the merging of several research fields, such as molecular biology, plant physiology, chemical ecology, and community ecology. In the end we will also rely on long-term field experiments for a complete understanding of the long term benefits and potential risk with the use of GM trees. Long-term field experiments in natural settings are essential for our understanding of the risks for secondary pest outbreaks or resistance evolution in pests, and are thus instrumental for developing efficient management practices that deal with these and other potential problems related to the use of GM trees. In addition, long-term field experiments are also essential for ecological safety assessments of GM trees (e.g., potential effects of non-target species or important ecosystem processes) and thus for our ability to balance benefits and risks with GM trees.

### Conflict of interest statement

The authors declare that the research was conducted in the absence of any commercial or financial relationships that could be construed as a potential conflict of interest.
